# An outbreak of hemolytic uremic syndrome associated with antibiotic treatment of hospital inpatients for dysentery.

**DOI:** 10.3201/eid0104.950407

**Published:** 1995

**Authors:** S Al-Qarawi, R E Fontaine, M S Al-Qahtani

**Affiliations:** Saudi Arabian Field Epidemiology Training Program, Ministry of Health, Saudi Arabia.


### An Outbreak of Hemolytic Uremic Syndrome Associated with Antibiotic Treatment of Hospital Inpatients for Dysentery

Shiga toxin (ST) from *Shigella dysenteriae* type 1 is accepted as a cause of hemolytic uremic syndrome (HUS); however, the reasons why HUS develops in only some infected patients are not clear (1). The possibility that antibiotic therapy is associated with the development of HUS has been explored for *S. dysenteriae* type 1 and for *Escherichia coli* O157:H7 (2–4). In May 1993, during an outbreak of *S. dysenteriae* type 1 in Gizan, Saudi Arabia, an association between antibiotic treatment and HUS was also observed (5). The strain of *S. dysenteriae* type 1 was resistant to ampicillin, tetracycline, chloramphenicol, and trimethoprim-sulfamethoxazole (TMP-SMX) and sensitive to nalidixic acid. We report here some of our preliminary observations for a concurrent outbreak.

In response to the Gizan outbreak, a circular was sent to all regions of Saudi Arabia requesting immediate reports of *S. dysenteriae* type 1. One region reported three cases of *S. dysenteriae* type 1 with the same antibiotic resistance pattern. The patients were visitors from the Najran region, on the Yemen border. The affected family reported that many other persons in their community of Barshash, home of about 6,000 Yemeni immigrants, had recently had dysentery. The regional hospital in Najran confirmed that several local children had been treated for HUS, and an investigation was initiated.

Beginning in February 1993, the numbers of dysentery patients at the Barshash Primary Health Care Center began to increase from 6 patients per week to 110 in mid May. After control measures were initiated, weekly incidence decreased to zero by late June. According to policy, the Primary Health Care Center treated dysentery patients with oral rehydration, whereas those admitted to the regional hospital received antibiotics. At the regional hospital, *S. dysenteriae* type 1 was isolated from four Barshash residents and one resident of another community.

Between March and May, the illness of 10 (of 42) children admitted to the regional hospital for dysentery progressed to HUS from 2 to 10 days (median 5) after admission. For all 10 children, physicians noted HUS onset as a sudden change in the clinical condition characterized by pallor, puffy face, peripheral edema, or oliguria. Within 2 days of clinical onset, blood urea nitrogen (BUN) levels of all 10 children were 10 mmol/L. In nine children, the hematocrit fell to below 75% of its value on admission. One child had microcytic hypochromic anemia on admission and was transfused with one unit of packed red cells; his hematocrit fell from 29.8% after the transfusion to 24.6% the day after HUS onset; his BUN level rose from 3.1 to 23.5 mmol/L; and his thrombocyte count fell from 529,000/$mm^3$ to 75,000/$mm^3$. Red cell fragments were reported for seven patients. Thrombocytopenia, developed in seven patients; in two, thrombocyte counts decreased from admission values. A leukemoid reaction (≥ 30,000 leukocytes/$mm^3$) developed in seven patients.

HUS cases included six children from Barshash, one from a contiguous district, one with relatives in Barshash, and two visiting from southern Gizan region where the concurrent outbreak was occurring (5). No child was admitted from the community with HUS. Reasons given for admission were either dysentery or bloody diarrhea with mild or moderate dehydration. Two dysentery patients who developed HUS were admitted because no one was available to care for them at home. Characteristics on hospital admission of dysentery patients who did or did not develop HUS were similar with the exception of serum sodium level and BUN (Table 1). Elevations in creatinine (63 to 133 mmol/L) and urea (6.1 to 7.9 mmol/L) levels on admission were mild, and after intravenous rehydration returned to normal. No enteric pathogens were isolated in stool specimens from 35 patients.

All 42 dysentery patients received a variety of antibiotic combinations composed of ampicillin, TMP-SMX, nalidixic acid, gentamicin, erythromycin, and metronidazole. Higher rates of HUS were observed among patients receiving antibiotics to which the locally circulating *S. dysenteriae* type 1 was resistant (ampicillin or TMP-SMX) or antibiotics that are ineffective against *Shigella* (metronidazole, erythromycin, gentamicin) than

Table 1. Characteristics on hospital admission of dysentery patients whose illness did or did not develop into hemolytic uremic syndrome (HUS) during hospitalization, Najran, Saudi Arabia, March through May 1993

| Characteristic | Dysentery patients: Developed HUS (10) | Dysentery patients: Did not develop HUS (32) | *p* value[a] |
|---|---|---|---|
| Age (yr mean) | 4.4 | 4.8 | 0.71 |
| Male sex | 7 (70%) | 23 (72%) | 1.0 |
| Percentile weight for age[b] (median) | 15.4 | 16.3 | 0.57 |
| Below fifth percentile | 6 (60%) | 12 (38%) | 0.29 |
| Days with dysentery (median) | 3 | 3 | 0.70 |
| Range | 2–8 | 1–14 | |
| Stools on first hospital day (median) | 12.5 | 9.0 | 0.23 |
| Range | 2–26 | 2–43 | |
| Temperature (mean) | 38.2°C | 38.0°C | 0.64 |
| Range | 36.8°C–39.4°C | 36.5°C–39.4°C | |
| Hematocrit (mean) | 38.9% | 38.8% | 0.94 |
| Below 35.5% | 4 (40%) | 7 (23%) | 0.43 |
| Thrombocytes/$mm^3$ | 555,000 | 501,000 | 0.62 |
| Below 150,000 | 0 | 0 | |
| Leucocytes/$mm^3$ (mean) | 11,440 | 12,300 | 0.62 |
| Above 12,000 | 7 (70%) | 22 (69%) | 1.0 |
| Above 18,000 | 1 (10%) | 4 (13%) | 1.0 |
| Serum sodium, mmol/L (mean) | 130 | 134 | 0.02 |
| Below 130 mmol/L | 4 (40%) | 7 (22%) | 0.41 |
| Serum potassium, mmol/L (mean) | 3.4 | 3.4 | 0.86 |
| Below 3.5 mmol/L | 5 (50%) | 16 (50%) | 1.0 |
| Serum creatinine, mmol/L (mean) | 72 | 62 | 0.62 |
| Above 62 mmol/L | 5 (50%) | 13 (41%) | 1.0 |
| BUN, mmol/L (mean) | 4.1 | 3.2 | 0.06 |
| Above 6.0 mmol/L | 2 (20%) | 2 (6%) | 0.24 |

[a]Differences in means by Student's t-test, in medians by the Kruskal-Wallis test, and in proportions by Fisher's exact test (two-tailed). [b]National Center for Health Statistics.

among patients treated with nalidixic acid (with or without metronidazole) (Table 2). Stratification of this analysis by elevated creatinine level (62 mmol/L) or BUN (6.0 mmol/L) on admission or by weight for age above and below the fifth percentile had no effect on the magnitude or statistical significance of these associations. However, stratification by serum sodium on admission above and below 130 mmol/L yielded increased risk ratios of 15 (95% confidence interval, 1.6 to 147) for any ineffective antibiotic and 15 (95% confidence interval, 2.3 to 99) for ampicillin without nalidixic acid (Mantel-Haentzel analysis).

These strong associations of HUS with prior antibiotic therapy suggest that the antibiotics may be influencing the progression of *S. dysenteriae* type 1 to HUS (2,5). The Najran patients had milder disease on admission, and the risk ratios were higher than in the other two reports. The assumption that *S. dysenteriae* type 1 caused the dysentery is supported by the isolation of *S. dysenteriae* type 1 from community members during a concomitant dysentery outbreak and by the absence of other ST producing organisms. However, because culture results are lacking, it is possible that some hospitalized dysentery patients were infected with other organisms.

This investigation was retrospective, and without randomization, the selection of patients for treatment by severity of illness was managed with stratified analysis. All possible indicators of dehydration or severity of dysentery on admission were not available for stratification. However, these were also not available to the physicians for selection of patients. Moreover, nalidixic acid was considered effective therapy, and physicians did not indicate in the medical records that they were selecting nalidixic acid for less severely ill patients. Because of the variety of antibiotics prescribed, the effect of antibiotics given to most patients (metronidazole) in combination with other antibiotics could not be evaluated. The effectiveness of those given to a few patients (TMP-SMX) could not be assessed.

Table 2. Risk of developing hemolytic uremic syndrome (HUS) by antibiotic combinations used for in-hospital treatment of dysentery during a community outbreak of antibiotic-resistant *Shigella dysenteriae* type 1, Najran, Saudi Arabia, March through May 1993

| | Dysentery patients | | | | |
|---|---|---|---|---|---|
| Antibiotic combination | Developed HUS (10) | Total (42) | HUS rate/100 | Risk ratio[a] | 95% confidence interval[b] |
| Ineffective[c] antibiotics without nalidixic acid[d–j] | 6 | 12 | 50 | 4.3 | 1.3–15 |
| Ampicillin combinations without nalidixic acid[d–f] | 5 | 7 | 71 | 6.2 | 1.9–20 |
| Ineffective antibiotics without nalidixic acid or ampicillin[g–j] | 1 | 5 | 20 | 1.7 | 0.22–14 |
| Ampicillin and nalidixic acid with (2) or without (3) metronidazole | 1 | 3 | 33 | 2.9 | 0.40–20 |
| Trimethoprim-sulfamethoxazole, nalidixic acid and metronidazole | 0 | 1 | 0 | 0 | |
| Nalidixic acid with (24) or without (2) metronidazole (reference) | 3 | 26 | 12 | 1.0 | Reference |

[a] Relative to the reference antibiotic combination (nalidixic acid with or without metronidazole). [b] Taylor series approximation standard. [c] Antibiotics to which the outbreak strain of *S. dysenteriae* type 1 was resistant (ampicillin, trimethoprim-sulfamethoxazole) or which are ineffective against shigella (metronidazole, gentamicin, or erythromycin). [d] Ampicillin and metronidazole (4 patients). [e] Ampicillin, metronidazole, and gentamicin (2 patients). [f] Ampicillin only (1 patient). [g] Trimethoprim-sulfamethoxazole and metronidazole (1 patient). [h] Trimethoprim-sulfamethoxazole, erythromycin, and metronidazole (1 patient). [i] Metronidazole only (2 patients). [j] Erythromycin and metronidazole (1 patient).

Antibiotic therapy may be associated with HUS in various ways. The antibiotics given may have been ineffective, so the infections were allowed to run their natural course to HUS. However, one would have expected at least a few cases to have developed at home among the hundreds of Barshash residents with dysentery. Another possibility is that ineffective antibiotics suppressed competing microbial flora, allowing less restrained proliferation of *S. dysenteriae* type 1. This does not account for the 12% HUS attack rate in patients treated with nalidixic acid. An in-vitro study of enhanced Shiga-like toxin I (SLT-1) production by *E. coli* O157:H7 suggests another explanation. Subinhibitory concentrations of antibiotics resulted in up to a 400% increase in SLT-I recovery relative to controls (6). This effect was maximal at maximum subinhibitory concentrations of antibiotics, and a quinoline antibiotic, ciprofloxacin, yielded the greatest increase of toxin. These findings are most consistent with the epidemiologic findings in this outbreak, including the occurrence of HUS in patients treated with nalidixic acid and the absence of HUS in untreated patients in the community. With these considerations, antibiotic treatment of dysentery from *S. dysenteriae* type 1 should be approached with caution.

**Sami Al-Qarawi,* Robert E. Fontaine,† and Mohammed-Saeed Al-Qahtani***
*Saudi Arabian Field Epidemiology Training Program, Ministry of Health, Saudi Arabia; †Epidemiology Program Office, Centers for Disease Control and Prevention, Atlanta, Georgia, USA.

### References

1. Moake JL. Haemolytic uremic syndrome: basic science. Lancet 1994;343:393-401.
2. Butler T, Islam MR, Azad MAK, Jones PK. Risk factors for development of hemolytic uremic syndrome during shigellosis. J Pediatr 1987;110:894-7.
3. Ostroff SM, Kobayahi JM, Lewis JH. Infections with *Escherichia coli* O157:H7 in Washington State: the first year of statewide disease surveillance. JAMA 1989;262:355-9.
4. Pavia AT, Nichols CR, Green DP, et al. Hemolytic-uremic syndrome during an outbreak of *Escherichia coli* O157:H7 infections in institutions for mentally retarded persons: clinical and epidemiologic observations. J Pediatr 1990;116:544-51.
5. Bin Saeed AAA, El Bushra HE, Al-Hamdan NA. Does treatment of bloody diarrhea due to *Shigella dysenteriae* type 1 with ampicillin precipitate hemolytic uremic syndrome? Emerging Infectious Diseases 1995;4:134-8.
6. Walterspiel JN, Ashkenazi S, Morrow AL, Cleary TG. Effect of subinhibitory concentrations of antibiotics on extracellular shiga like toxin I. Infection 1992;20:25-9.
